# Post-traumatic stress disorder following childbirth

**DOI:** 10.1186/s12888-021-03158-6

**Published:** 2021-03-16

**Authors:** Deniz Ertan, Coraline Hingray, Elena Burlacu, Aude Sterlé, Wissam El-Hage

**Affiliations:** 1 Université de Lorraine, CNRS, CRAN, UMR 7039, Nancy, France; 2 La Teppe, Tain l’Hermitage, France; 3 Pôle Hospitalo-Universitaire de Psychiatrie d’Adultes du Grand Nancy, Centre Psychothérapique de Nancy, Laxou, France; 4 CHRU de Tours, Centre Régional de Psychotraumatologie CVL, Tours, France; 5 UMR 1253, iBrain, Université de Tours, Inserm, Tours, France

**Keywords:** Post-partum post-traumatic stress disorder, Childbirth, Mother-infant bond, Trauma

## Abstract

**Background:**

Childbirth experience could be complicated and even traumatic. This study explored the possible risk factors for post-traumatic stress disorder following childbirth (PTSD-FC) in mothers and partners.

**Methods:**

Through a cross-sectional online survey biographical, medical, psychological, obstetrical and trauma history data were collected. The PTSD-FC, postnatal depression, social support, and perceived mother-infant bond in 916 mothers and 64 partners were measured through self-reported psychometric assessments.

**Results:**

Our findings highlight the possible impact of several risk factors such as emergency childbirth, past traumatic experiences and distressing events during childbirth on PTSD-FC. The difficulties in mother-infant bond and the postpartum depression were highly associated with the total score of PTSD-FC symptoms for mothers. While for partners, post-partum depression was highly associated with the total score of PTSD-FC.

**Conclusions:**

Our study demonstrated significant links between psychological, traumatic and birth-related risk factors as well as the perceived social support and the possible PTSD following childbirth in mothers and partners. Given that, a specific attention to PTSD-FC and psychological distress following childbirth should be given to mothers and their partners following childbirth.

## Introduction

Childbirth could be experienced as distressing or even traumatic for some women, which might produce undesirable marks on their lives. A traumatic childbirth could cause psychological distress, intense fear, or helplessness for the parturient and increases the risk of anxiety, depression and even post-traumatic stress disorder (PTSD) [1, 2]. One study showed that about 45% of women experienced traumatic childbirth [3] and up to 4–6% of women developed PTSD following childbirth (PTSD-FC) [4, 5]. Women who experience PTSD-FC might feel abandonment, guilt and helplessness. These feelings have direct impact on mother-child interactions and could cause important social isolation [6]. Moreover, couples relationships could be negatively affected by a traumatic childbirth experience and PTSD-FC symptoms [7]. During the postpartum, women could suffer from mental health disorders related to birth experience. The risk of postpartum depression, postpartum psychosis, and anxiety are increased after a complicated childbirth [8–10]. Moreover, postpartum is associated with increased risk of suicide, especially for women who suffer from post-partum depression [11]. One study showed that one of the non-medical leading causes of death for women during the postpartum is suicide [12]. In France, suicide is the second cause of post-partum death during 2013–2015 [13].

Several studies examined the risk factors for developing PTSD-FC [14–19]. Negative experiences and severe fear of childbirth [14], subjective distress [15], previous abortion [16], psychological difficulties in pregnancy (particularly depression in early pregnancy) [17], previous psychiatric problems [14], history of PTSD and trauma (particularly interpersonal violence) [14] are possible psychological risk factors for developing PTSD following childbirth. Several obstetric and birth-related factors such as pregnancy complications (e.g., labour and obstetrical emergencies) [15, 19], modes of birth (e.g., emergency caesarean) [14, 19] could also contribute to PTSD-FC in women. Additionally, different environmental factors like poor interaction between provider and mother [18], low social support during labour and birth [14, 18, 19] are associated with development of PTSD-FC.

The partners could also be affected by the childbirth experience. One recent study showed that unexpected events during pregnancy and the childbirth could cause a “rollercoaster of emotion” in partners [20]. On one hand, partners who feel more prepared for the possible complications during pregnancy and childbirth, who feel supported and included to the procedure have more positive experiences of childbirth [21]. On the other hand, partners who feel excluded, abandoned, unconfident during pregnancy and childbirth process, are more likely to develop distress and avoidance coping strategies [20]. Unplanned pregnancies, being less prepared, and higher trait anxiety are the possible risk factors for negative emotional experiences after childbirth in partners [20, 22].

Birth trauma experiences do not affect only mothers but they also affect the relationship of mothers with their partners. Negative childbirth experiences have important short- and long-term consequences on the couples relationships and on the parent-child interactions [23, 24]. Indeed, the state of well-being following childbirth both in mothers and their partners is critical for the child’s development [25]. Therefore, the mental health following childbirth constitute an important subject of research. Even though PTSD-FC is a very important topic, there is few research focusing on the possible PTSD following childbirth in mothers and also in partners. Our aim was to explore psychological, traumatic and birth-related factors for possible PTSD-FC in women and the partners. The purpose of this study was to examine the link between history of traumatic events, child loss, modes of birth, distressing childbirth, social support, perceived mother-infant bond, postnatal depression and symptoms of PTSD-FC. We expect to find differences between the modes of birth, the history of traumatic events, and that there is a relationship between postnatal depression, social support, perceived mother-infant bond and PTSD-FC.

## Methods

### Procedure

We adopted a cross-sectional online questionnaire through a secure survey platform (Sphinx software), with a 9-month enrolment period. The study and consent procedures were approved by the ethics committee of the University (Comité d’Ethique de la Recherche Tours-Poitiers, n°2019-09-01). Online advertisement and snowballing were used to recruit participants. All participants endorsed an online informed consent and were subsequently directed to the online questionnaire. Measures were taken between 1-month to 12-months postpartum. Male or female participants completed the online survey assessing demographics, obstetric history, psychological history, birth experience, symptoms of anxiety, depression, PTSD, perceived parental bonding, and social support. Participants were instructed to answer about their experience during the birth of their most recent baby. They were asked about potential traumatic events during (or immediately after) the birth, and if they experienced post-traumatic symptoms related to this birth. The online procedure facilitated access to the study for a larger number of participants and also allowed participations based on the availability of subjects.

The exclusion criteria comprised: women who gave birth less than 1 month or more then 1 year ago; partners of women who gave birth less than 1 month or more than 1 year ago; persons under guardianship or curatorship; persons with difficulty to understand the informed consent form; participants who did not fill out the survey completely.

### Measures

Biographical data were collected using sociodemographic questions, medical, psychological, obstetrical history, and traumatic life events. A self-reported psychometric assessment allowed to measure the PTSD-FC, postnatal depression, social support, and perceived mother-infant bond.

*Sample description.* The information regarding obstetric and birth-related history were collected through several questions such as the number of pregnancies, the number of children, the sex of the last child, the mode of the last birth – emergency caesarean, vaginal vacuum, vaginal (no instrument) and vaginal (forceps). We also investigated the experience of distressing events during childbirth by asking “Have you experienced particularly upsetting or traumatic event during childbirth?” We also asked if the participants have lost a child before (i.e., miscarriage, stillbirth, etc.). Moreover, we explored the history of other traumatic events by asking the participants if they have experienced a traumatic event in their lifetime (e.g., accident, natural disaster, illness, unexpected death, violent attack, or sexual abuse). If yes, they were asked to give a brief description of the traumatic event.

The *City Birth Trauma Scale (CBTS)* is a self-reported questionnaire developed by Ayers et al. (4) in response to the need for an instrument for assessing PTSD following childbirth [26]. This scale includes 31 items, 29 of which correspond to the diagnostic criteria for PTSD according to the DSM-5 (including intrusion, avoidance, negative cognition and mood, and hyperarousal), as well as additional questions to assess the subjective criterion and symptoms of emotional insensitivity [26]. Of the 31 items, 23 of them are based on a Likert-type scale (0 = not at all, to 3 = 5 or more times). The questionnaire has two subscales to evaluate general symptoms and birth-related symptoms (score range 0–69). The response scale for symptoms asks for frequency of symptoms over the last week. The highest score reveals a higher risk for PTSD. An additional questionnaire for partners was also developed. It helps identifying women and partners with PTSD-FC. The psychometric properties have been evaluated and validated by Ayers et al. (4) and replicated in Hebrew [27], Croatian [28] and Turkish [29]. The CBTS has shown good psychometric properties to detect symptoms of PTSD-FC. According to the study of Ayers et al., the reliability analyses of CBTS showed high internal consistency (Cronbach’s α = .92) [26]. In this study, a non-validated French version of CBTS was used. We also performed reliability analysis for CBTS total and subscales. Similar to previous studies, our results showed high internal consistency for CBTS total scale (α = .92), for CBTS birth related symptoms (α = .90) and CBTS global symptoms (α = .91).

The *PTSD checklist for DSM-5 (PCL-5)* is a questionnaire developed by Weathers et al., according to the diagnostic criteria of the DSM-5 [30]. The scale is composed of 20 items assessing the intensity of the 20 criteria for PTSD symptoms presented in the DSM-5. Responders were invited to respond a Likert-type scale (0 = not at all, to 4 = extremely) to evaluate the level of bother that they felt for each item during the past month [30]. This self-questionnaire was validated for the screening and monitoring of PTSD and validated in French [31]. Scores higher than 33 indicate provisional diagnosis of PTSD. High internal consistency was demonstrated for the French version of PCL-5 (Cronbach’s α = .94) [31] and in the present study (α = .94).

The *Edinburgh Postnatal Depression Scale (EPDS)* is a 10-item scale developed by Cox et al. [32] translated and validated in French [33]. Responders were invited to score each item from 0 to 3, which allows to assess depressive symptoms in the post-partum period. The scores range from 0 to 30. Score more than 10 indicates provisional diagnosis of depression. The Cronbach’s coefficient of .76 was demonstrated for the French version of EPDS [33]. Our results showed high internal consistency (α = .886).

The *Medical Outcomes Study (MOS)* is a 20 item-scale developed by Sherbourne & Stewart [34]. The items are divided into 4 categories to evaluate different types of support (emotional, tangible, affectionate and positive social interactions). Responders were invited to score (0 = none of the time, to 5 = all of the time) for each item in order to evaluate how often these different types of supports are available. The internal consistency was found high for MOS in the study of Sherbourne & Stewart (Cronbach’s α = .91) [34] and very high in our study (α = .958).

The *Mother-to-Infant Bonding Scale (MIBS)* is an eight item-questionnaire developed by Taylor et al. [35]. Each item refers to an adjective to evaluate the participants feelings towards their baby during the first week that follows childbirth. The items are based on Likert-type scale (0 = not at all, to 3 = very much). The scores range between 0 and 24. This scale assesses the perceived difficulties of the mother-child bond during the first weeks of the newborn’s life. It was validated in French [36]. The scale was also used with fathers to assess their bonding scores with their child [37]. The internal consistency was demonstrated for MIBS as acceptable in previous studies (Cronbach’s α = .71) [36] and high in the present study (α = .824).

### Statistical analyses

Descriptive statistics were calculated for sociodemographic, medical, psychological, obstetrical characteristics as well as traumatic life events. The bivariate ANOVA tests were performed to test the group differences on the scores of CBTS total and subscales for mothers and for their partners separately (dependent variables). The tests were performed between groups who were either exposed to a potential risk factor or not (i.e., child loss, past traumatic experiences) or whether the different modes of birth differed (i.e., mode of birth, distressing childbirth). Regarding post hoc tests for ANOVA, we first performed multiple comparison between independent variables. We then performed the post hoc Tukey test when the Levene’s test for homogeneity of variances was non-significant, and the post hoc Games Howell test when the Levene’s test for homogeneity of variances was significant.

We also performed Bravais-Pearson correlations to calculate the bivariate associations between the MOS, the MIBS, the EPDS, the PCL-5, the CBTS total scale and subscales for mothers and for partners. The Bravais-Pearson correlations could allow us to explore the relationship between several biopsychosocial factors (i.e., depression, perceived social support, mother-infant bonding) and CBTS total and subscales (possible PTSD-FC). Descriptive and statistical analyses were performed using SPSS 24.0 software (IBM).

## Results

Of the 1093 entries, 980 participants completed the full questionnaire including all the assessments: 916 mothers and 64 partners were retained for analysis. Of the 916 mothers, 203 had a previous distressing childbirth, 465 had at least one traumatic event in their lives. Of the 64 partners, 7 had a previous distressing childbirth, and 23 had at least one traumatic event in their lives.

For the majority of the mothers, this was their first childbirth and majority of them had a vaginal birth. Postnatal depression measured using the EPDS revealed mean score for mothers above 10 points (below 10 for partners). The mothers to infant bond scale (MIBS) revealed low mean scores for mothers and for partners. The social support scale (MOS) mean score was found moderate in mothers and partners. The PTSD symptoms measured by the PCL-5 and the CBTS revealed mean scores below the cut-offs in both groups. The sample characteristics were presented in Table 1.

**Table 1:** Sample characteristics

	Mothers (***n*** = 916)	Partners (***n*** = 64)
Previous distressing childbirth, % (n)	22.16 (203)	10.9 (7)
Previous child loss, % (n)	24.24 (222)	15.6 (10)
Past traumatic event (excluding birth-related), % (n)	50.76 (465)	35.9 (23)
Type of birth, % (n)		
Vaginal	60.8 (556)	–
Emergency caesarean	16.7 (152)	–
Planned caesarean	4.3 (39)	–
Vaginal (forceps)	8.9 (81)	–
Vaginal (vacuum)	9.6 (88)	–
Number of children, % (n)		
1	61.3 (561)	–
2	28.8 (263)	–
3	9.0 (73)	–
4	1.2 (10)	–
5+	1 (9)	–
Sex of the baby, % (n)		
Twins	1.7 (15)	–
Boy	50.5 (463)	–
Girl	47.8 (438)	–
MOS, M (SD)	55.25 (14.9)	55.02 (17.27)
EPDS, M (SD)	11.79 (7.57)	8.48 (6.73)
MIBS, M (SD)	3.04 (3.4)	2.64 (3.28)
PCL-5, M (SD)	15.42 (15.55)	12.56 (13.8)
CBTS-BRS, M (SD)	5.68 (6.9)	2.36 (4.03)
CBTS-GS, M (SD)	20.59 (17.27)	5.86 (6.78)
CBTS-Total, M (SD)	16.68 (13.81)	8.25 (9.16)

*BRS* Birth-related symptoms, *CBTS* City Birth Trauma Scale, *EPDS* Edinburgh Postnatal Depression Scale, *GS* General symptoms, *MIBS* Mother-to-Infant Bonding Scale, *MOS* Medical Outcomes Study, *PCL-5* PTSD checklist for DSM-5

### For mothers

#### Correlation analyses

We performed a correlation analysis on the study dimensions. We performed Bravais-Pearson correlations between the scores of the MOS, the MIBS, the EPDS, the PCL-5, the CBTS total scale and subscales for mothers. All correlations were significant. Low social support (MOS) was significantly correlated with higher scores at the CBTS total scale, the CBTS general symptoms subscale and the CBTS birth-related symptoms subscale. We found positive correlations between perceived mother-child bond and the CBTS total scale, the CBTS general symptoms subscale and CBTS birth-related symptoms subscale. Similarly, postnatal depression (EPDS) was significantly correlated with PTSD-FC as evaluated by the CBTS total scale, the CBTS general symptoms subscale and the CBTS birth-related symptoms subscale. Lastly, we found significant concordant results between the PCL-5 scores and the CBTS total scale, the CBTS general symptoms subscale and the CBTS birth-related symptoms subscale (Table 2).

**Table 2:** Correlation analyses (coefficients r de Bravais-Pearson) for mothers

	MOS	MIBS	EPDS	PCL-5	CBTS-BRS	CBTS-GS	CBTS-total
MOS	1						
MIBS	−.126^a^	1					
EPDS	−.260^a^	.506^a^	1				
PCL-5	−.361^a^	.396^a^	.661^a^	1			
CBTS-BRS	−.218^a^	.345^a^	.530^a^	.667^a^	1		
CBTS-GS	−.351^a^	.366^a^	.585^a^	.773^a^	.491^a^	1	
CBTS-Total	−.340^a^	.412^a^	.648^a^	.839^a^	.819^a^	.901^a^	1

*BRS* Birth-related symptoms, *CBTS* City Birth Trauma Scale, *EPDS* Edinburgh Postnatal Depression Scale, *GS* General symptoms, *MIBS* Mother-to-Infant Bonding Scale, *MOS* Medical Outcomes Study, *PCL-5* PTSD checklist for DSM-5

^a^Correlation is significant at the .01 level (2-tailed); *ns* non-significant

#### One-way ANOVA tests

We performed bivariate ANOVAs to test the group differences between exposed and non-exposed mothers to a potential risk factor (i.e., trauma history, child loss), and whether the different modes of birth differed (i.e., mode of birth, distressing childbirth) on the scores of CBTS total and subscales. We found significant group differences for the mode of birth on CBTS birth-related symptoms (F (4,911) = 20.6, *p* < .001, η^2^ = .083), on CBTS general symptoms (F (4,911) = 4.1, *p* < .005, η^2^ = .018) and on CBTS total score (F (4,911) = 11.6, p < .001, η^2^ = .049). Women who had an emergency caesarean had the highest scores on CBTS total score and on CBTS birth-related subscale. Women who had vaginal birth with vacuum and women who had vaginal birth with forceps had the second and the third highest scores on CBTS total and on CBTS birth-related symptoms subscale. In contrast, women who had vaginal birth without instrument had the lowest scores on CBTS total score and on CBTS birth-related symptoms. For general symptoms subscale, women who had emergency caesarean and vaginal vacuum had the highest scores. In contrast, women who had vaginal birth without instrument and forceps birth had the lowest scores in CBTS general symptoms subscale. In regard to the CBTS-BR scores, post hoc tests showed that women who had vaginal birth differed significantly from women who had emergency cesarean (*p* < .001, d = .791), vaginal vacuum (*p* < .002, d = .552), and forceps birth (*p* < .018, d = .477), while women who had emergency cesarean differed from women who had programmed cesarean (*p* < .001, d = .608). We found a medium effect size (η^2^ = .083), so we can assume that 8.3% of the variance in CBTS-BR was a result of the type of birth. For the CBTS-GS scores, only women who had vaginal birth differed significantly from women who had emergency cesarean (*p* < .014, d = .297). There was a small effect size for the different modes of birth in regard to the CBTS-GS scores (η^2^ = .018). Regarding the total score of CBTS, women who had vaginal birth differed significantly from women who had emergency cesarean (*p* < .001, d = .589) and vaginal vacuum (*p* < .011, d = .469). For this ANOVA test, we found a small effect size (η^2^ = .049).

We also found a significant group differences for distressing events during childbirth on CBTS birth-related symptoms (F (1,914) = 106.5, *p* < .001, η^2^ = .104), on CBTS general symptoms (F (1,914) = 16.5, *p* < .001, η^2^ = .018) and on CBTS total scale (F (1,914) = 60.3, *p* < .001, η^2^ = .062). Women who had distressing events during childbirth had higher scores on CBTS total score and subscales compared to women who did not experience distressing events during childbirth. We found no significant group difference for history of child loss on the CBTS total scale and subscales. In contrast, we found a significant group differences for experiencing past traumatic events on CBTS general symptoms (F (1,914) = 6.8, *p* < .01, η^2^ = .007) and on CBTS total scale (F (1,914) = 8.4, *p* < .005, η^2^ = .009). Women who experienced past traumatic events had higher scores on CBTS general symptoms subscale and on CBTS total scale. However, no such difference was found for CBTS birth-related symptoms (F (1,914) = 5.636, *p* = .018).

### For partners

#### Correlation analyses

We also conducted the same correlation analysis on the study dimensions for partners. We found no significant correlation between social support (MOS) and CBTS total scale and subscales were found. In contrast, we found a positive correlation between perceived mother-child bond (MIB) and CBTS general symptoms as well as CBTS total symptoms. However, there were no significant correlation between MIB and CBTS birth-related symptoms. We found positive correlations between postnatal depression (EPDS) and CBTS total scale, CBTS birth-related symptoms and CBTS general symptoms. Consistently, PCL-5 symptoms were positively correlated to CBTS total scale, CBTS birth-related symptoms and CBTS general symptoms (Table 3).

**Table 3:** Correlation analyses (coefficients r de Bravais-Pearson) for partners

	MOS	MIBS	EPDS	PCL-5	CBTS-BRS	CBTS-GS	CBTS-total
MOS	1						
MIBS	−.387^a^	1					
EPDS	−.385^a^	.527^a^	1				
PCL-5	−.449^a^	.514^a^	.797^a^	1			
CBTS-BRS	-.234^*ns*^	.133^*ns*^	.523^a^	.542^a^	1		
CBTS-GS	-.162^*ns*^	.378^a^	.397^a^	.420^a^	.395^a^	1	
CBTS-Total	-.223^*ns*^	.338^a^	.524^a^	.550^a^	.733^a^	.914^a^	1

*BRS* Birth-related symptoms, *CBTS* City Birth Trauma Scale, *EPDS* Edinburgh Postnatal Depression Scale, *GS* General symptoms, *MIBS* Mother-to-Infant Bonding Scale, *MOS* Medical Outcomes Study, *PCL-5* PTSD checklist for DSM-5

^a^Correlation is significant at the .01 level (2-tailed); *ns* non-significant

#### One-way ANOVA tests

We performed bivariate ANOVAs to test for group differences between exposed and non-exposed partners to a potential risk factor (i.e., trauma history, child loss), and whether the different modes of birth differed (i.e., mode of birth, distressing childbirth) on the scores of CBTS total and subscales. We found no significative results regarding CBTS total score for child loss (F (1,62) = .101, *p* = .757) or distressing childbirth (F (1,62) = 1.654, *p* = .203), but the differences in past trauma scores were significant (F (1,62) = 5.149, *p* = .027, η^2^ = .077).

## Discussion

Our aim was to explore psychological, traumatic and birth-related factors for possible PTSD-FC in women and the partners. Our findings highlight group differences concerning several risk factors such as emergency childbirth, past traumatic experiences and distressing events during childbirth on the development of PTSD following childbirth in mothers. Likewise, our results shows that the PTSD-FC are correlated positively with difficulties in mother-infant bond, scores of depression and correlated negatively with perceived social support.

### Traumatic factors

Similar to earlier studies [14, 38–43], our results showed that PTSD-FC symptoms were higher in mothers who priorly experienced a traumatic event compared to mothers who did not. For instance, a systematic review of risk factors for childbirth induced PTSD showed that previous exposure to trauma is an important risk factor for developing PTSD following childbirth [14]. Experiencing two or more traumatic events [43], history of sexual trauma [40, 41], traumatic experiences during childhood [38] or childhood maltreatment [42] could increase the likelihood of developing PTSD following childbirth. These results showed that previous traumatic experiences have multiple roles during postpartum period by increasing the odds of future PTSD for mothers. One study highlighted the importance of resilience as a protective factor against possible PTSD-FC [42]. Mothers who are at greater risk for developing PTSD-FC could benefit from resilience-enhancing interventions [42]. Our results also showed that partners who experienced past traumatic events have significantly higher scores in CBTS total scale compared to partners who did not experience past traumas. These results highlight the past traumatic events as a factor increasing the likelihood of possible PTSD following childbirth in partners. Mental health of both parents is crucial for child’s development. It is therefore important to be vigilant about the traumatic background of mothers but also of their partners, while preparing for childbirth.

### Birth-related factors

According to our results women who experienced childbirth as distressing had higher scores in PTSD-FC. We also found a significant group difference for the mode of birth on the scores of PTSD-FC. We found that women who had emergency caesarean were more vulnerable for developing PTSD-FC. Our findings are consistent with literature. According to several studies, birth complications and mode of birth are the factors associated with potential PTSD-FC [44–46]. Women who had emergency caesarean had higher risk of developing PTSD-FC compared to women who had vaginal birth [45, 46]. According to a recent systematic review [19] emergency caesarean and labour pain are identified risk factors for negative childbirth experiences for women. Similar to emergency caesarean, several studies highlighted the risk for developing PTSD after a major surgery in adults and in children [47–49]. It is therefore important to give a particular attention to patients for possible distress and PTSD after major or emergency surgeries, to identify those who test positive and refer them for treatment, such as trauma-focused psychotherapy.

### Biopsychosocial factors

We also examined the relationship between PTSD-FC and social support, PTSD symptoms, the quality of child-mother bond and the postnatal depression by focusing on birth-related PTSD symptoms and general PTSD symptoms in mothers and in partners. Our results demonstrated that mothers who had higher score in PCL-5 had also higher scores in CBTS total and subscales. These results highlights that the general PTSD symptoms are correlated with PTSD following childbirth.

Our results demonstrated that the difficulties in perceived child-mother bond and the postpartum depression were highly associated with birth-related PTSD symptoms, general PTSD symptoms and total score of PTSD-FC symptoms for mothers. Several studies showed a negative impact of PTSD on mother-infant relationship [50–53]. According to Davies et al. [50], mothers with PTSD symptoms have more negative maternal representations and they describe their babies as less warm and more invasive. Similarly, Parfitt & Ayers [51] found that symptoms of PTSD and depression have a negative impact on couples relationships as well as parent-infant bond. According to a recent study [53], PTSD and depressive symptoms have negative impact on infant-mother bond. However, in contrast to our findings, the authors did not find a correlation between the birth-related PTSD symptoms with mother-infant bonding [53]. Although, the authors demonstrated an indirect effect of general PTSD symptoms on mother-infant bonding via depressive symptoms [53].

Our results showed a positive correlation between depression and PTSD-FC symptoms for mothers. Several studies showed a high comorbidity between postnatal depression and PTSD following childbirth [23, 54] In this light, Söderquist et al. [17] suggested that depression and PTSD might share the common vulnerabilities and risk factors. In addition, several studies showed that the depression is a significant predictor for post-partum PTSD symptoms [55].

We found a negative correlation for the social support and the symptoms of PTSD-FC for mothers. These results showed the importance of social support during pre- and post-partum period for mothers. One meta-analysis found that poor social support is one of the risk factors associated with PTSD-FC [54]. According to this study, the lack of social support in general is a vulnerability factor for developing PTSD-FC while the lack of support from medical staff is one of the risk factors during birth for PTSD-FC. Likewise, several studies showed the absence of social support associated with higher risk for developing PTSD symptoms following childbirth [41, 56, 57]. A recent systematic review [19] found that perceived control during labour as well as the strong partner support are the important protective factors to have a positive childbirth experience for mothers. We did not find a relationship between social support and PTSD-FC for partners.

Similar to mothers, we found that post-partum depression was highly associated with birth-related PTSD symptoms, general PTSD symptoms and PTSD-FC for partners. Consistent with our results, one study found a significant relationship between depressive symptoms and symptoms of PTSD (avoidance, intrusion, hyperarousal) in partners of women who gave birth. Another study found a positive correlation between depression and PTSD scores for partners who experienced pregnancy complications caused by preterm preeclampsia or preterm premature rupture of membranes [58].

We also found that partners who had more difficulties in parent-child bonding, had also higher scores in general PTSD symptoms and in PTSD-FC. Several studies showed that higher symptoms of depression and anxiety caused a poorer parent-baby interaction [51, 59]. Likewise, according to previous scientific literature, the symptoms of PTSD and depression associated with lower quality of parent-baby bond [23, 51].

### Limitations

The current study has several limitations. First, the data of this study was obtained from an online survey and therefore our results should be interpreted with caution. Second, our findings for partners were limited by our small sample size. For this reason, our study population might not be representative. Overall, these limitations reveal some concerns about the generalizability of our data. The correlation analysis gave an important overview to understand the positive and negative associations between several variables, however it did not provide a causal relationship. It is therefore important to complete this study with further researches. The CBTS is a promising questionnaire to assess the PTSD-FC; however, the French versions of the CBTS were not yet validated, therefore it is important to interpret our results cautiously although reliability analysis in this study was promising. The current research was based on a cross-sectional retrospective study, therefore we remain prudent in the interpretation of our results. Further and larger longitudinal studies are needed to confirm our results. Thus, further studies are required to validate the French versions of the CBTS for mothers and partners, replicate our findings in a different population sample of women and at different time points across the postpartum period, include a larger sample of partners, and use structured diagnostic interviews in order to confirm our results using different methodological approaches.

## Conclusion

Our study demonstrated significant links between psychological and traumatic risk factors as well as the perceived social support and PTSD following childbirth in mothers and partners. Given that, we should pay specific attention to possible PTSD following childbirth in mothers but also in their partners. A systematic screening for possible risk factors for developing PTSD-FC might be a beneficial approach to identify the most vulnerable mothers. Moreover, it is also important to inform mothers and their partners sooner rather than later for the possible complications during pregnancy and delivery in order to prepare them for pregnancy and childbirth. Therefore, it is important to conduct further studies that focus on potential PTSD following childbirth with the ultimate goal of identifying early interventions to implement on maternal and paternal PTSD-FC.

## Data Availability

The datasets used and/or analysed during the current study are available from the corresponding author on reasonable request.

## Abbreviations

PTSD-FC: Post-traumatic stress disorder following childbirth
PLC-5: PTSD checklist for DSM-5
EPDS: Edinburgh Postnatal Depression Scale
MOS: Medical Outcomes Study
MIBS: Mother-to-Infant Bonding Scale
CBTS: City Birth Trauma Scale
BRS: Birth-related symptoms
GS: General symptoms
